# Cerebrospinal fluid exosomal protein alterations via proteomic analysis of NSCLC with leptomeningeal carcinomatosis

**DOI:** 10.1007/s11060-023-04428-x

**Published:** 2023-09-01

**Authors:** Lan Hou, Xin Chen, Gang Qiu, Xuejiao Qi, Yueli Zou, Junying He, Hui Bu

**Affiliations:** 1 Department of Neurology, The Second Hospital of Hebei Medical University, 215 Heping West Road, Shijiazhuang, 050000 China; 2 Department of Neurology, Baoding No.1 Central Hospital, Baoding, China; 3 Secondary Department of Oncology, Hebei General Hospital, Shijiazhuang, China

**Keywords:** Leptomeningeal carcinomatosis, Cerebrospinal fluid, Exosome, Proteomics, Parallel reaction monitoring

## Abstract

**Purpose:**

Leptomeningeal carcinomatosis (LC) is a rare complication of non-small cell lung cancer (NSCLC) with highly mortality. Cerebrospinal fluid (CSF) as a special kind of tumor microenvironment (TME) better represents alterations than plasma. However, the clinical value of protein profiles of exosome in CSF as liquid biopsy remains unclear.

**Methods:**

In this study, CSF samples of NSCLC patients with (LC group) or without (NSCLC group) LC were collected and compared to patients without tumors (normal group). CSF exosomes were isolated by ultracentrifugation and protein profiles were performed by label-free proteomics. Differentially expressed proteins (DEPs) were detected by bioinformatics tools and verified by parallel reaction monitoring (PRM).

**Results:**

A total of 814 proteins were detected. Bioinformatics analysis revealed their shared function in the complement activation, extracellular region, and complement and coagulation cascades. Between LC and NSCLC group, 72 DEPs were found among which FN1 demonstrated the highest betweenness centrality (BC) after protein-protein interaction network analysis.

**Conclusion:**

We investigated the application of label free and PRM based proteomics to detect key proteins related to LC. FN1 may serve as potential indicator to classify LC and NSCLC. Extracellular matrix (ECM) and epithelial-mesenchymal transition (EMT) are important in the process of LC. These data is promising for early prediction and diagnosis of LC.

**Supplementary Information:**

The online version contains supplementary material available at 10.1007/s11060-023-04428-x.

## Introduction

Leptomeningeal carcinomatosis (LC) happens when cancerous cells reach the leptomeninges, subarachnoid space, and other cerebrospinal fluid (CSF) compartments [1]. The most common origin is the lung [2]. LC is a rare complication of non-small cell lung cancer (NSCLC). Only 3–5% of patients with advanced non-small cell lung cancer (NSCLC) are associated with LC [3]. The median survival period is usually short [1], and little is known about the underlying mechanisms. Adrienne Boire et al. demonstrated that cancer-cell-derived C3 had the ability to disrupt the blood-CSF barrier and facilitated the tumor-related plasma components, therefore promoted cancer cell growth [4]. Besides, cancer cells appeared to survive in the CSF by outcompeting macrophages for iron [5].

The tumor microenvironment (TME) is composed of cancerous, non-cancerous, stromal, and immune cells that are surrounded by components of the extracellular matrix (ECM) [6]. TME plays a critical role in the proliferation and invasion of tumor cells, especially in central nervous system metastases. The cellular and molecular mechanisms have been reported in several studies [7, 8]. When leptomeningeal metastases happen, tumor cells infiltrate the blood-CSF barrier and circulate within the CSF which is regarded as a special TME for LC patients.

Exosomes (exos), derived from different cells, are lipid bilayer spheroids with a diameter of about 30-200 nm [9]. Many types of molecules are present in exos, including proteins, lipids, and nucleic acids [10]. Exos are key components in TME. As an important cell-to-cell way of communication, exos participate in a variety of physiological and pathological processes. Tumor cell-derived exos demonstrated the ability to interact with ECM to create a microenvironment suitable for tumor growth, resulting in distant tumor metastasis [11]. Lung cancer cell-derived extracellular vesicles could target endothelial cells, altering the permeability of the blood-brain barrier by affecting actin, resulting in brain parenchymal metastasis [12]. In comparison to peripheral blood exos, little is known about the functional roles of CSF exos in the context of LC.

Considering proteins as the main executors of biological functions, in this study proteins in CSF exos were gathered and analyzed by label-free proteomics and bioinformatics tools. Parallel reaction monitoring (PRM) was performed to verify the proteins related to LC.

## Methods

### Study design

**Fig. 1:**
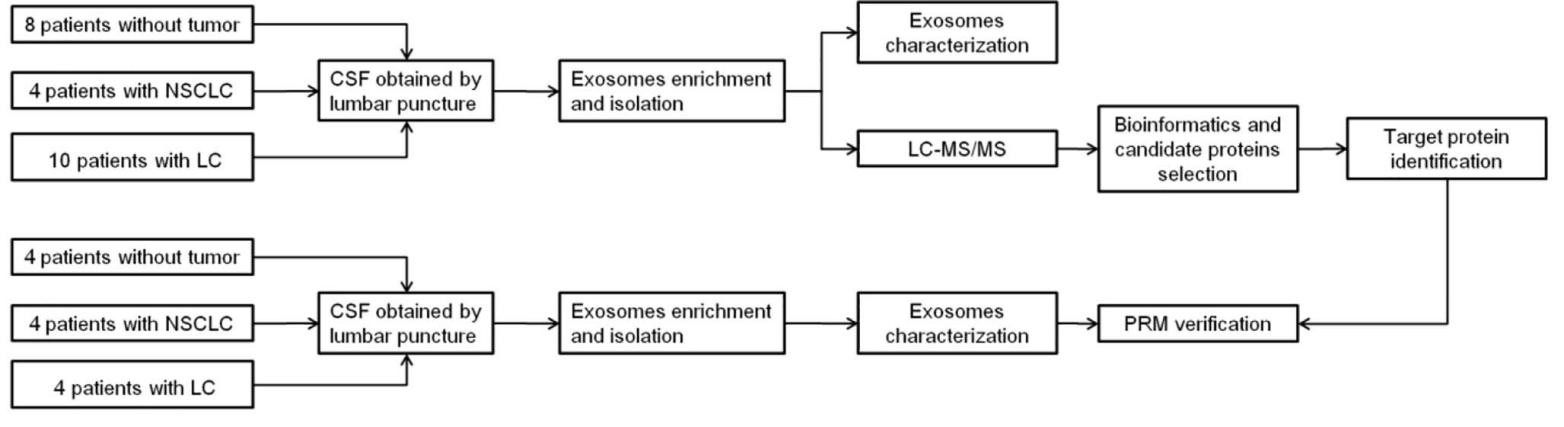
Workflow of study design. NSCLC, non-small cell lung cancer; LC, leptomeningeal carcinomatosis; LC-MS/MS, liquid chromatography with tandem mass spectrometry; PRM, parallel reaction monitoring

A work flow was shown in Fig. 1. Subjects were divided into three groups, named LC group, NSCLC group and normal group. Patients in LC and NSCLC groups suffered NSCLC conformed by pathology. LC was determined by established criteria based on CSF cytology and neuroimaging evaluation [13]. Normal group was defined as subjects without tumor evidence. Their clinical and laboratory parameters were recorded and CSF samples were collected.

### CSF sample collection

CSF samples were obtained in the Second Hospital of Hebei Medical University (Shijiazhuang, China) by lumbar puncture according to the standard protocol [14]. All patients provided written informed consents before sample collection. All the protocols were approved by the Ethics Committee of Clinical Research of the Second Hospital of Hebei Medical University. CSF samples after routine examination were collected. Then they were centrifuged at 3,000 g for 15 min to remove the cells inside. The supernatant was aliquoted into 1.0 mL eppendorf tubes and stored at -80 °C. All procedures were performed at 4 °C within 30 min after CSF obtainment.

### CSF exos enrichment and isolation

**Fig. 2:**
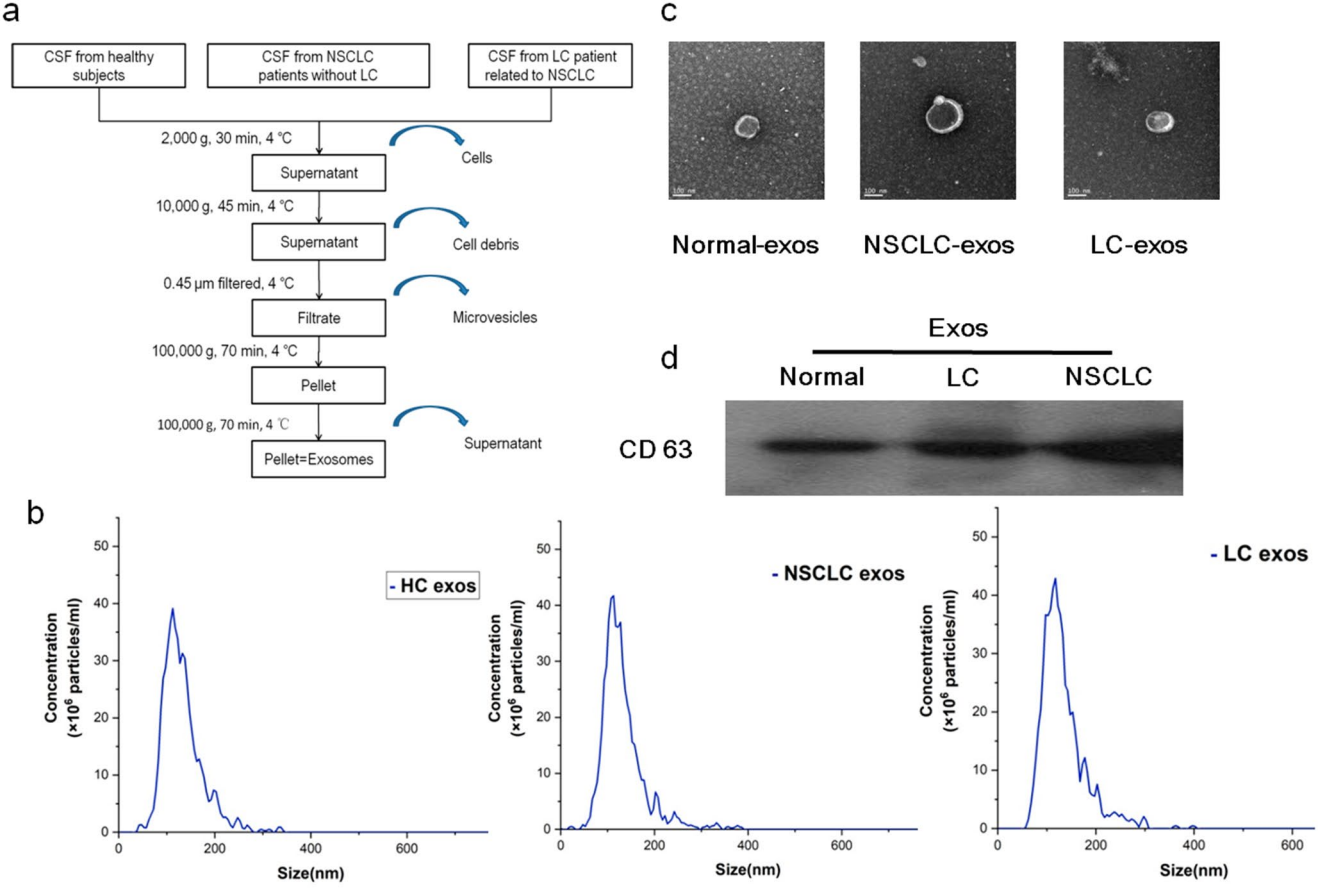
Isolation and verification of CSF exos. **a** Isolation of CSF exos by ultracentrifugation. **b** Size distribution of exos via NTA analysis. **c** TEM images of exos derived from three groups (Scale bar 100 nm). **d** Exos expressions of CD 63 by Western blotting analysis. CSF, cerebrospinal fluid; exos, exosomes; NSCLC, non-small cell lung cancer; LC, leptomeningeal carcinomatosis; NTA, nanoparticle tracking analysis; TEM, transmission electron microscopic

Exos were isolated by a centrifugation method reported previously with minor modifications [15] as shown in Fig. 2a. Briefly, CSF samples were rapidly thawed at 4 °C. Because the remaining CSF of a single patient is little, CSF was mixed to three samples in each group. Then they were moved to a new tube and centrifuged two consecutive steps at 2,000 g (30 min) and 10,000 g (45 min) at 4 °C to remove cells. Supernatant was filtered with 0.45 μm polyvinylidene difluoride (PVDF) membrane. Filtrate was transferred to a clean tube followed by ultracentrifuged at 100,000 g for 70 min. Pellets were re-suspended and washed with cold phosphate buffered saline (PBS) followed by additional ultracentrifugation at 100,000 g for 70 min at 4 °C. The final pellet was collected and stored.

### Nanoparticle tracking analysis

Nanoparticle tracking analysis (NTA) was used to measure the concentration and size of exos by Particle Tracking Analyzer (ZetaView PMX 110, Particle Metrix, Germany). The analyzer was calibrated with polystyrene microspheres (110 nm) and washed with PBS. Each sample was analyzed at least for three times by scanning 11 detection positions each. The NTA software was used to analyze the obtained data.

### Transmission electron microscopic

The morphological characteristics of exos were detected by transmission electron microscopic (TEM). In brief, 20 uL PBS suspensions of exos were loaded onto Formvar/carbon-coated TEM grids for 20 min at room temperature. The excess solutions were removed and a volume of 20 uL 2% phosphotungstic acid was loaded for 20 s. The images of exos were captured by JEOL (Jem-1400, Japan).

### Western blotting analysis

10 µg proteins of LC exos sample were mixed with 4X loading buffer and run in 10% gel. Then they were transferred to a polyvinylidene difluoride (PVDF) membrane, which was subsequently incubated with primary antibodies: CD 63 (ab 216,130, Abcam). They were then incubated with a secondary antibody (7074, CST). Chemiluminescent imaging was carried out by Tanon 4600 Imaging System (Shanghai, China).

### Protein extraction and trypsin digestion

Dithiothreitol (Sigma) was added to 30 µL of sample to the final concentration of 100 mM, bathed in boiling water for 5 min and cooled to room temperature. Then it was mixed well with 200 µL of UA buffer (8 M Urea, 150 mM Tris-HCl, pH 8.5), transfered to a 30 kD ultrafiltration centrifuge tube and centrifuged at 12,500 g for 15 min twice. Iodoacetamide buffer (100 mM iodoacetamide in UA) was used as a decontaminant at room temperature in darkness. 40 mM ammonium bicarbonate was added to reduce the urea concentration of dithiothreitol. Finally, the proteins were digested with trypsin buffer (4 µg trypsin in 40 µL 40 mM NH4HCO3 solution) for 16–18 h. The digested peptides were desalted with C18 cartridge, lyophilized and reconstituted with formic acid solution. Then they were quantified under OD 280.

### Liquid chromatography with tandem mass spectrometry (LC-MS/MS) analysis

Sample was separated by an Easy nLC system (Thermo Fisher Scientific, Waltham, MA, USA). Phase A solvent was 0.1% formic acid aqueous solution, and phase B solvent was 0.1% formic acid acetonitrile aqueous solution (with acetonitrile 80%). The column was equilibrated with 100% solvent A, and the sample was loaded by an autosampler onto an analytical column (Thermo Fisher Scientific, Acclaim PepMap RSLC 50 μm X 15 cm, nano viper, P/N164943) for separation at a flow rate of 300 nL/min. The peptides were chromatographed and analyzed by tandem mass spectrometry in Q Exactive Plus (Thermo Fisher Scientific, Waltham, MA, USA). The m/z scan range was 350 to 1800, and intact peptides were detected in the Orbitrap at a resolution of 70,000.

### Bioinformatics analysis

Maxquant software package (1.5.5.1) was used to retrieve the secondary MS data, and the Swiss-Prot_Human data was used as reference. The sequences of the proteins were mapped according to their Gene Ontology (GO) based on the DAVID database (https://david.ncifcrf.gov/home.jsp). Protein-enriched pathways assessment was performed by the Kyoto Encyclopedia of Genes and Genomes (KEGG) database. The identified proteins were compared with available exos data from ExoCarta database (http://www.exocarta.org). To reveal the potential interactions among the differentially expressed proteins (DEPs), protein-protein interaction (PPI) networks were constructed using the STRING: functional protein association networks tools (http://string-db.org). The interaction networks of the proteins with high confidence (combined_score ≥ 0.4) were established in Cytoscape. Visualization and network analyses were performed in Cytoscape (v 3.8.0). The betweenness centrality (BC) of the protein network was analyzed by the CytoNCA function of Cytoscape.

### PRM analysis

According to the previous inclusion criteria, 4 patients in each group were enrolled in PRM analysis. After exos separation and proteolysis, concentration of exosomal protein was listed in supplementary Table 4. PRM detection and analysis were performed. About 2 µg peptide was taken from each sample for detection. After chromatographic separation, samples were analyzed by mass spectrometry with Q-Exactive Plus (Thermo Fisher Scientific). Analysis duration: 60 min, detection mode: positive ion. Primary mass spectrometry scanning range: 350–1800 m/z, mass spectrometry resolution: 70,000, AGC target: 3e6 and Maximum IT: 200 ms. The original files were analyzed by software Proteome Discoverer 2.1.

### Statistical analysis

Student’s t-test was used to compare the statistical difference between groups. Proteins were regarded as DEPs when the fold changes were equal to or higher than 2 and false discovery rate-adjusted P value was less than 0.05. P value < 0.05 was considered statistically significant.

## Results

### Study cohort

8 patients with cerebral venous sinus thrombosis, idiopathic intracranial hypertension and headache were in normal group. 14 patients, previously diagnosed with NSCLC, were suspected LC. 4 of them were excluded after CSF, neuroimaging and clinical evaluation and included in the NSCLC group. The other 10 patients were in the LC group. Details of patients in the LC group were shown in Table 1.

**Table 1:** Details of patients in leptomeningeal carcinomatosis group

Patient No.	Age	Sex	Tumor cells in cytology	ICP (mmH_2_O)	CSF protein (g/L)	Other metastatic sites	CSF gene testing	Mutation sites
1	62	M	Yes	>500	0.10	Bone	-	
2	54	F	Yes	>500	0.30	Bone	Yes	EGFR/ CDKN2A/GNAQ/KDR
3	46	F	Yes	200	0.20	-	Yes	EGFR/TP 53
4	47	F	Yes	350	0.36	-	-	
5	66	F	No	450	0.48	Bone	Yes	TP 53
6	53	M	Yes	140	0.42	Bone/liver	Yes	EGFR/TP 53
7	43	F	Yes	>500	0.10	Bone	Yes	EGFR/TP 53
8	56	M	Yes	110	0.40	-	Yes	EGFR
9	53	M	Yes	450	0.77	None	-	
10	82	F	Yes	400	0.13	-	-	

Abbreviations: No., number; ICP, Intracranial pressure; CSF, cerebrospinal fluid

### Biochemical and morphological characterizations of CSF exos

Exos were evaluated for biochemical, morphological and molecular characteristics (Fig. 2). The particle size distribution in the normal group was between 42.5 and 337.5 nm, with most particles demonstrating a 121.5 nm diameter (Fig. 2b) and the particle number was 5.7 × 10^8^ particles/ml. The particle number was 6.9 × 10^8^ particles/ml and the peak diameter was 118.6 nm (22.5- 382.5 nm) in the NSCLC group (Fig. 2b). Meanwhile, the particle number was 6.2 × 10^8^ particles/ml in the LC group and the peak diameter was 119.3 nm (62.5- 397.5 nm) (Fig. 2b). Typical cup-shaped and round vesicles were observed by TEM and representative TEM images were shown in Fig. 2c. All the three groups were confirmed the presence of surface markers CD 63 (Fig. 2d).

### Proteomics characteristics of exosomal proteins

**Fig. 3:**
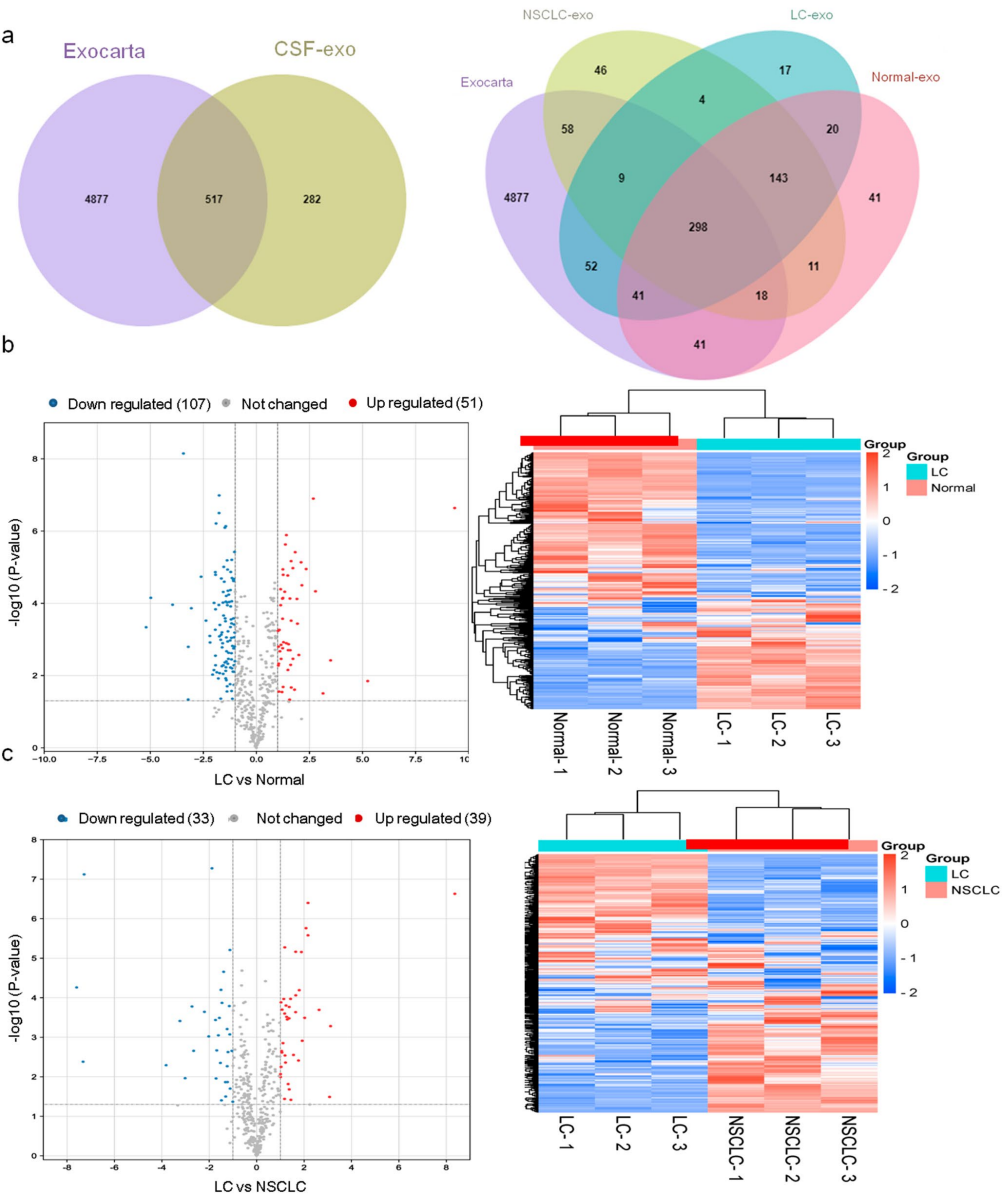
CSF exosomal label-free proteomics and DEPs between different groups. **a** Venn diagram of exosomal proteins in different groups compared with ExoCarta. **b** Volcano plots and cluster analysis of DEPs between LC and normal group. **c** Volcano plots and cluster analysis of DEPs between LC and NSCLC group. CSF, cerebrospinal fluid; Exo, exosome; NSCLC, non-small cell lung cancer; LC, leptomeningeal carcinomatosis; DEPs, differential expressed proteins

Most of the peptides were composed of 8–20 amino acids, qualified of the quality control requirements for LC-MS/MS analysis. Almost 64.3% of the proteins matched ExoCarta, and their unique 291 proteins enriched the existing exosomal library (Fig. 3a). A complete proteomic analysis of all the CSF-sourced exos revealed 7314 peptides and 814 proteins including 613, 587, and 585 proteins in normal, NSCLC, and LC exos, respectively (Fig. 3a). The mass spectrometry proteomics data have been deposited to the ProteomeXchange Consortium (http://proteomecentral.proteomexchange.org) via the iProX partner repository with the dataset identifier PXD039102.

The GO analysis classified the results as biological process, cellular component, and molecular function. In these three groups, biological process mapped to complement activation (Supplementary Fig. 1a, 2a and 3a), as well as cellular components including extracellular region (Supplementary Fig. 1b, 2b and 3b). In the molecular function, the identified proteins were significantly enriched in extracellular matrix structural constituent and endopeptidase inhibitor activity in normal CSF exos (Supplementary Fig. 1c). In terms of NSCLC and LC group, exos proteins related to molecular function were mainly involved in antigen binding/endopeptidase inhibitor activity (Supplementary Fig. 2c and 3c). As for the KEGG pathway analysis, it was showed that all exosome proteins were enriched in complement and coagulation cascades (Supplementary Fig. 1d, 2d and Fig. 3d).

### Identification and bioinformatics analysis of DEPs

We then compared the differentially expressed exosomal proteins in the LC group and the other two groups respectively (LC vs. normal and LC vs. NSCLC). In LC vs. normal comparison, there were 158 DEPs, 51 up-regulated and 107 down-regulated (Fig. 3b); in LC vs. NSCLC comparison, there were 72 DEPs, 39 up-regulated and 33 down-regulated (Fig. 3c). According to the hot map, protein expression is similar in the same group while different between different groups.

Focusing on LC, proteins that differentially expressed both between LC vs. normal and LC vs. NSCLC were summarized in Supplementary Table 1. Proteins in column 1, 3 and 5, totally twenty proteins, were chosen. After PPI analysis, the 4 top proteins with the highest BC were ACTB, ENO1, TIMP1 and RTN4R (Supplementary Fig. 4).

PPI analysis was performed between LC and the other two groups. Between LC and NSCLC group, the top protein with the highest BC was FN1 and the other proteins were shown in Supplementary Fig. 5. As to LC and normal group, the 20 top proteins with the highest BC were APP, ACTB, NCAM1, APOB, PRNP, CDH2, SPP1, CRP, FGA, PIGR, CTSD, HP, APLP1, ENO2, AGT, CNTN2, SOD1, TIMP1, ITIH3 and NRXN1 (Supplementary Fig. 6).

### Validation of DEPs by PRM

**Fig. 4:**
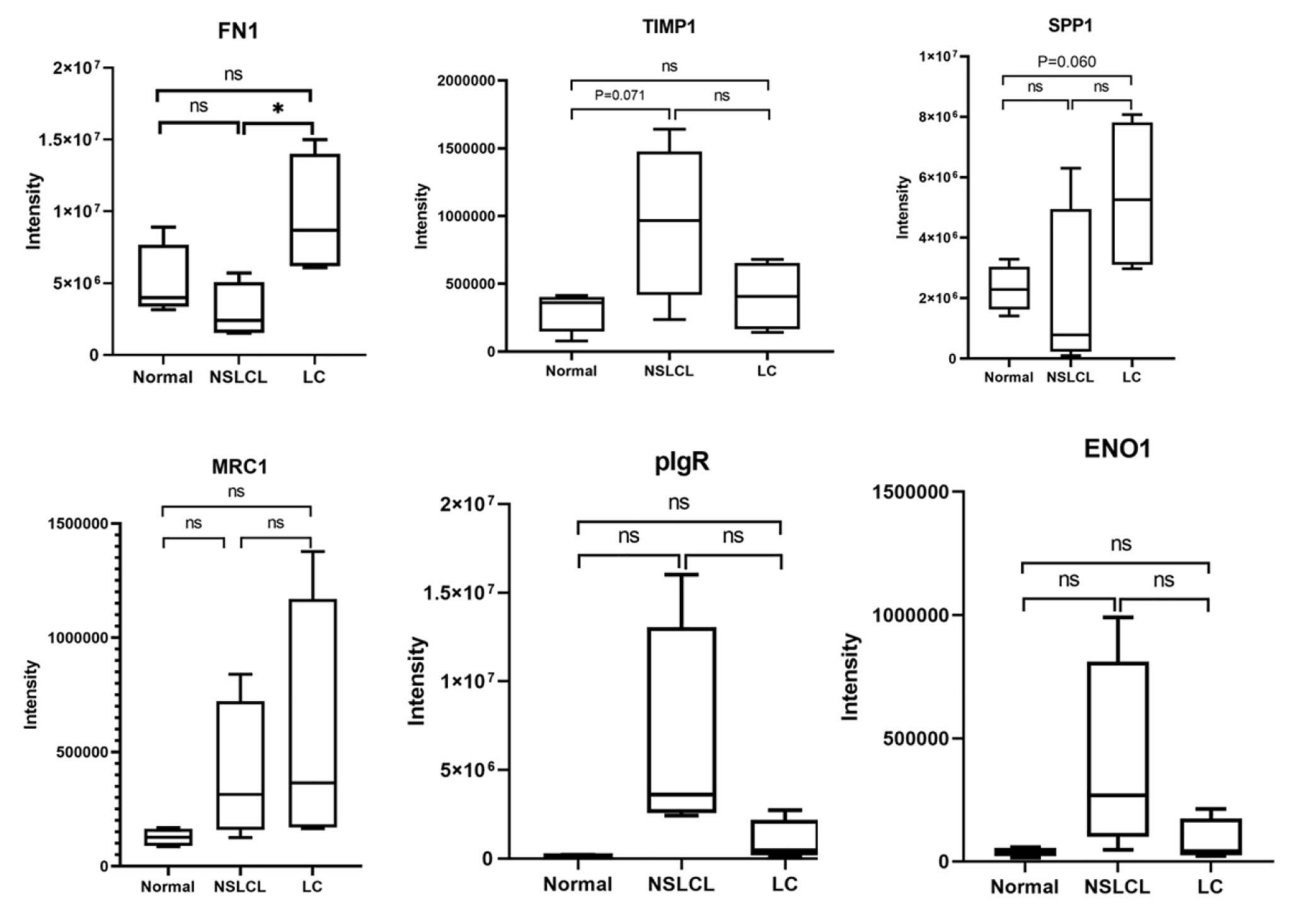
Quantitative results of proteins after parallel reaction monitoring

Based on previous researches, bioinformatics analysis, and clinical practice, we selected ENO1, TIMP1, SPP1, PIGR, FN1, and MRC1 as candidate proteins for subsequent validation. Target peptides suitable for PRM analysis were shown in Supplementary Table 2. Results were shown in Fig. 4 and Supplementary Table 3. FN1 was significantly higher in the LC group than in the NSCLC group (P = 0.029) which is consistent with proteomics. TIMP1 was higher in the NSCLC group than in the normal group while there was no statistical difference (P = 0.071). Compared with the normal group, SPP1 in the LC group was up-regulated, but there was no statistical difference (P = 0.060). Relations of survival and protein expression intensity were shown in Supplement Fig. 7.

## Discussion

Due to the restrictions of the blood-brain barrier, many therapeutic drugs cannot enter the central nervous system. Although great efforts have been made, it is still showed an extremely poor prognosis of the disease [16]. The prognosis of patients can be improved with an accurate and timely diagnosis. Targeted therapy, especially osimertinib, has shown promise in multiple clinical trials for LC treatment [16–20]. Therefore, it is of great importance to detect possible diagnostic biomarkers or therapeutic targets.

Liquid biopsy, comparing with tissue biopsy, has shown a significant step forward in the diagnosis of tumor because of its less invasive, lower cost, and real-time insights [21]. In general, liquid biopsies are divided into three major categories based on the tumor-derived materials namely circulating tumor DNA (ctDNA), circulating tumor cells (CTCs) and exos/extracellular vesicles. ctDNA [22, 23] and exosomal microRNAs [24] have shown aspects in the diagnosing and monitoring of LC. Further more, CSF seems more comprehensive than plasma in liquid biopsy of patients with LC because it carries more driver genomic mutations and potential prognostic markers [23].

Extracellular vesicles can pass through the blood-brain barrier and shuttle between the central nervous system and peripheral circulation [25]. It is promising both in the diagnosis and treatment of LC. Huiying Li et al. demonstrated upregulated hsamiR-509-3p and downregulated hsa-miR-449a may serve as potential indicators of intrathecal anti-pemetrexed [24]. The abundance of CSF proteins is low, making it difficult to obtain exos from CSF especially with an ultracentrifugation method. In this study, we report a comprehensive dataset of CSF exosomal proteins isolated by ultracentrifugation and identified by proteomics. It was demonstrated that exos in different groups were similar in size, morphological and molecular characteristics, which was consistent with previous reports [9, 26].

In this study, we for the first time used label free approach in conjunction with PRM analysis to perform a comprehensive profile of the CSF exosomal proteins among LC, NSCLC and normal group. Our proteomics analysis identified 814 proteins in CSF exos. This was similar with prior CSF exosomal protein isolated by ultracentrifugation [27, 28]. Proteomics revealed 64.3% of the proteins matched the existing exosomal library and the unique 291 proteins enriched the library. The identified proteins included proteins related to exos (CD9, CD81). Besides, several markers of neurons (NFASC, L1CAM and synaptic proteins NPTXs, NRXNs), astrocytes (ALDH1L1), myeloid cells (MMP2, MRC1), endothelial cell (ICAM, VCAM, VWF), epithelial cell (LAMA, LAMB), BBB (AGRN, DAG1, FBLNs), and CP (CALM) were also detected. This reflected the multiple origins of exos. Several oncogenes, for example, ENO1 and PIGR, were up-regulated in LC group. Besides, MRC1 (also known as CD206), which is recognized as marker of pro-tumoral M2 macrophage, was also up-regulated in LC group. We speculate that CSF of LC patients might be an immunosuppressive microenvironment with high expression of oncogenes.

Fibronectin is a kind of glycoprotein widely expressed by multiple cell types and involved in cell adhesion, migration, wound healing, coagulation and defense. Cancerous FN is highly expressed in the CTCs that possess a high metastatic potential empowered by the WNT signaling [29]. Fibronectin 1 (FN1) is referred as biomarkers for epithelial-mesenchymal transition (EMT) or ECM and facilitates tumor growth and metastasis. Activating the FN1 signaling may enhance the metastatic seeding of tumor cells in the lung [30]. FN1 is also known to play regulatory roles in NSCLC brain metastasis [31]. When epithelial cells obtain the mesenchymal phenotype, disruption of intercellular tight junctions and blood-brain barrier will facilitate cell migration [32]. FN1 was detected among LC and NSCLC group by combination of label free and PRM based proteomics and bioinformatics analysis. The impact of high stromal FN expression may be due to the induction of matrix metalloproteinases (MMPs) in tumor cells, which finally facilitates tumor migration, invasion, angiogenesis, and intravasation [33, 34].

In addition, tissue inhibitor of metalloproteinase-1 (TIMP1) and secreted phosphoprotein 1 (SPP1) identified in this study have certain potential biomarker capabilities. TIMPs are a natural inhibitor of MMPs which are a major group of proteases known to regulate the turn-over of ECM [35]. ECM remodelling is the result of an imbalance in the equilibrium of the normal processes of synthesis and degradation of ECM components markedly controlled by the MMPs/TIMP imbalance [36]. TIMP1 was up-regulated in NSCLC [35, 37]. SPP1 is a secreted glycoprotein with multifunction such as immune regulation, cell survival, and tumor progression. SPP1 is closely related to tumor progress, such as proliferation, migration, and invasion. Studies have demonstrated that SPP1 plays an important role in certain individual tumors [38]. The expression of SPP1 in lung adenocarcinoma tissues and cells was significantly higher than that in normal tissues and cells and demonstrated positive correlation with TNM stage, lymph node metastasis, and invasion depth and negative correlation with survival [39]. SPP1 facilitates cell migration and invasion by up-regulating COL11A1 expression [39]. Furthermore, down-regulation of SPP1 could reduce the expression of epithelial marker and increase the expression of mesenchymal markers. SPP1 is related to EMT.

### Limitations

This is the first study to integrate label free and PRM approach intended to figure out potential biomarkers related to LC. It seems that EMT and ECM may play important roles in LC. However, there are some limitations with regard to the research methods. Firstly, because of the difficulty of CSF sample collection, the sample size is small. Therefore, selection bias must be considered. Secondly, clinical value of these biomarkers failed to be obtained due to the small sample size. Large prospective study could provide more details to determine values of these LC related proteins.

## Conclusion

We investigated the application of label free and PRM based proteomics to detect key proteins related to LC. FN1 may serve as potential indicator to classify LC and NSCLC. Extracellular matrix (ECM) and epithelial-mesenchymal transition (EMT) are important in the process of LC. These data is promising for early prediction and diagnosis of LC.

### Electronic supplementary material

Below is the link to the electronic supplementary material.

Supplementary Material 1

Supplementary Material 2

## Data Availability

Datasets generated during this study are available from the corresponding author on reasonable request.
