# Supplementary material for: Cerebrospinal fluid exosomal protein alterations via proteomic analysis of NSCLC with leptomeningeal carcinomatosis
Source: J Neurooncol. 2023 Sep 1;164(2):367–76. doi: 10.1007/s11060-023-04428-x (PMC10522761; doi:10.1007/s11060-023-04428-x)
Supplement: Supplementary file 1 — Supplementary Material 1 [file 11060_2023_4428_MOESM1_ESM.docx]

**Supplementary Figure 1:** Bioinformatics analysis of normal group exosomes.


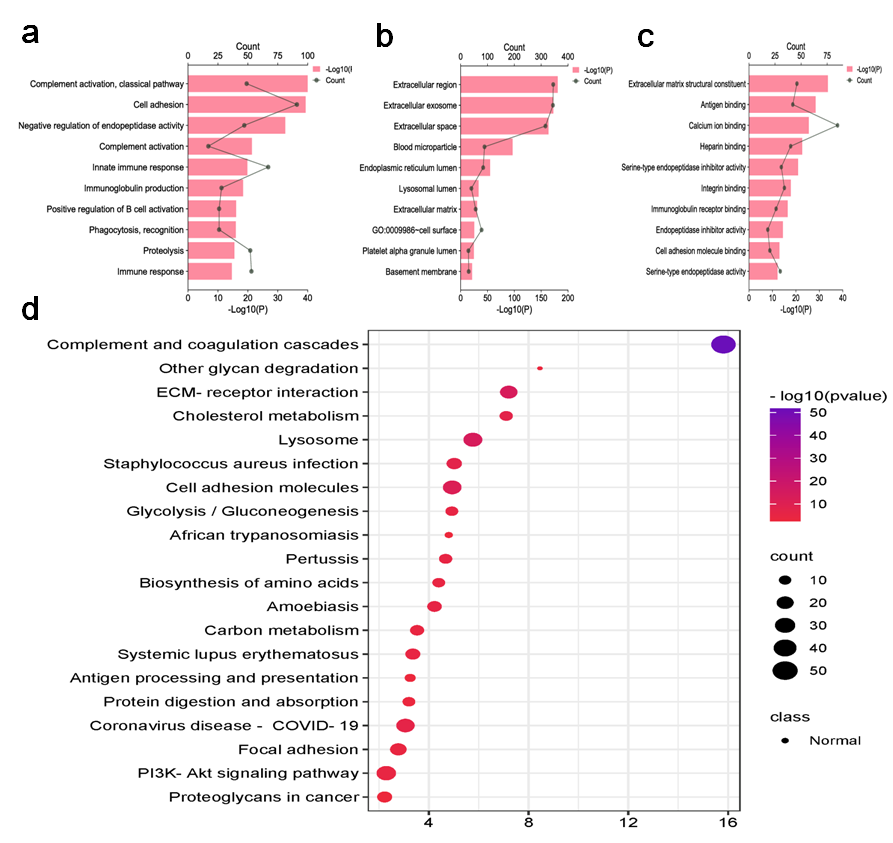


**a** Biological process of GO analysis. **b** Cellular component of GO analysis. **c** Molecular function of GO analysis. **d** KEGG analysis of exosomes.

**Supplementary Figure 2:** Bioinformatics analysis of non-small cell lung cancer group exosomes.


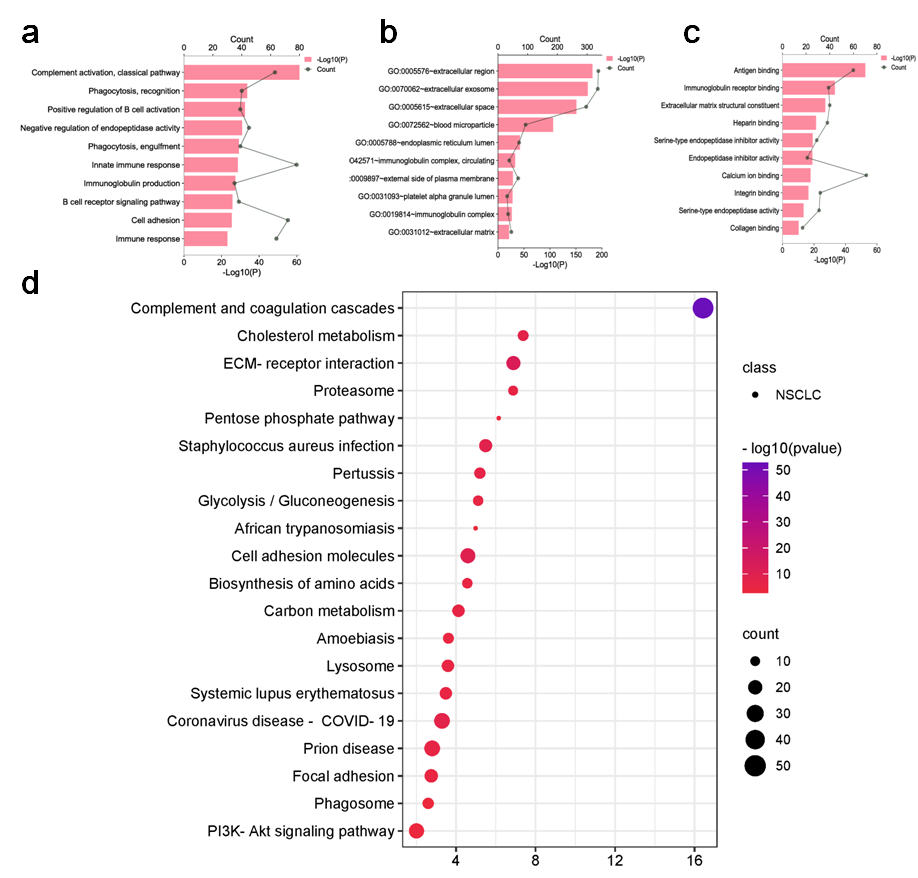


**a** Biological process of GO analysis. **b** Cellular component of GO analysis. **c** Molecular function of GO analysis. **d** KEGG analysis of exosomes.

**Supplementary Figure 3:** Bioinformatics analysis of leptomeningeal carcinomatosis group exosomes.


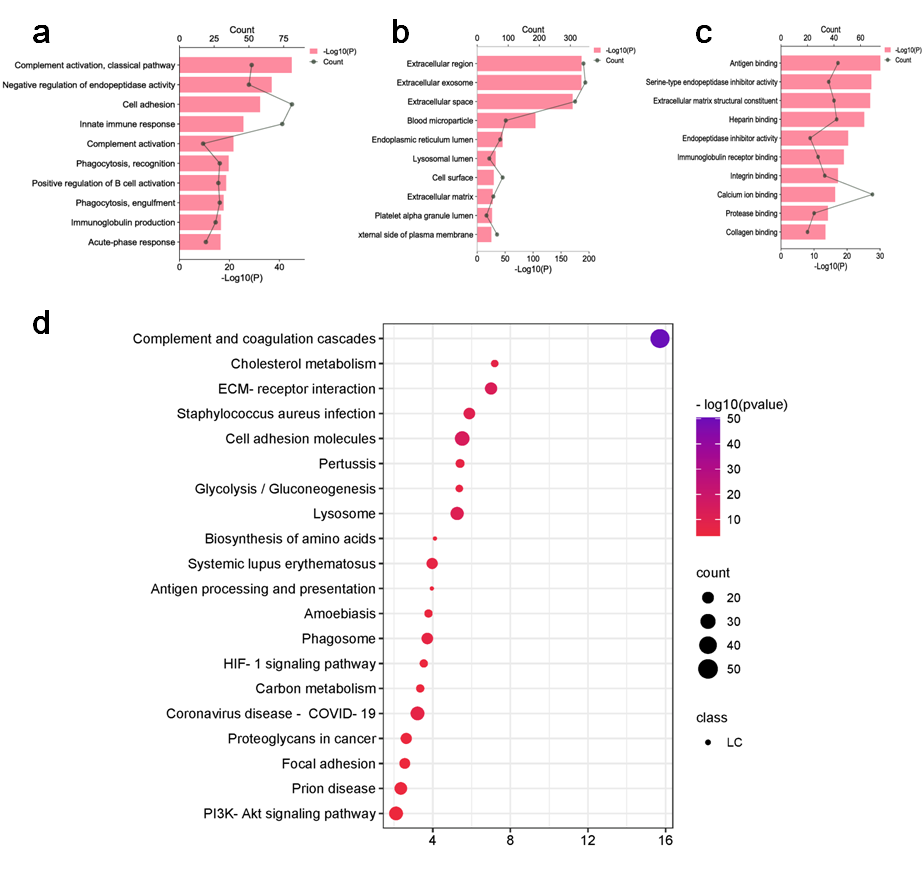


**a** Biological process of GO analysis. **b** Cellular component of GO analysis. **c** Molecular function of GO analysis. **d** KEGG analysis of exosomes.

**Supplementary Figure 4** Protein-protein interaction analysis of 20 DEPs related to leptomeningeal carcinomatosis.

**
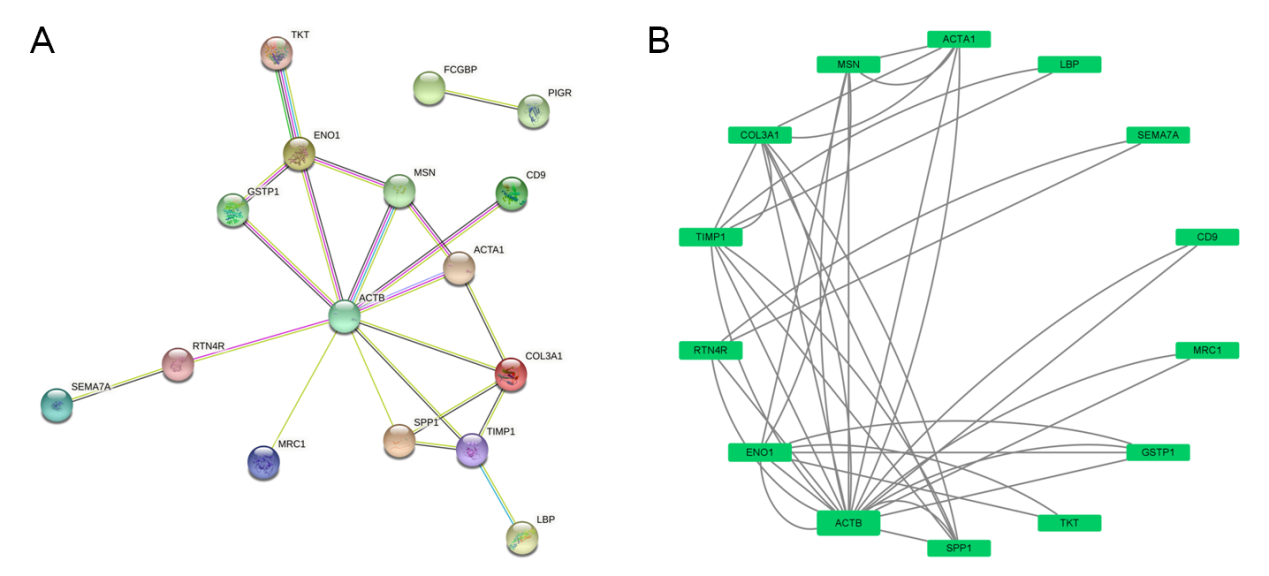
**

**Supplementary Figure 5** Protein-protein interaction analysis of DEPs between leptomeningeal carcinomatosis and non-small cell lung cancer groups.

**
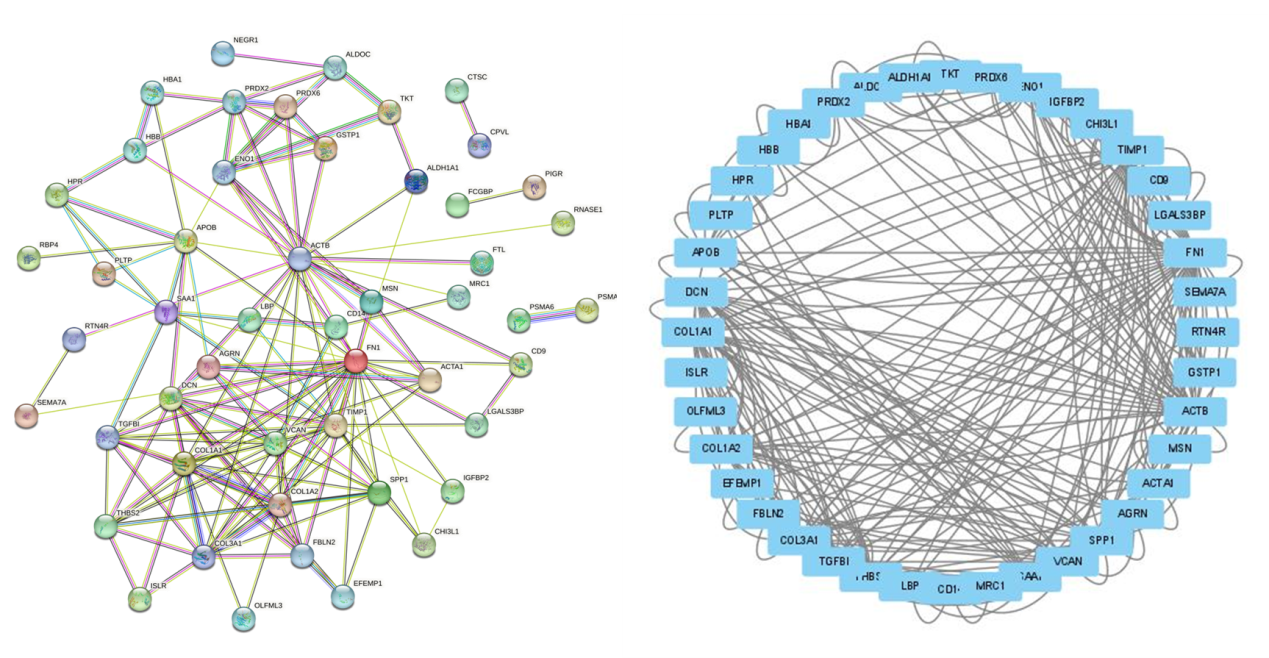
**

**Supplementary Figure 6:** protein-protein interaction analysis of DEPs between leptomeningeal carcinomatosis and normal groups.

**
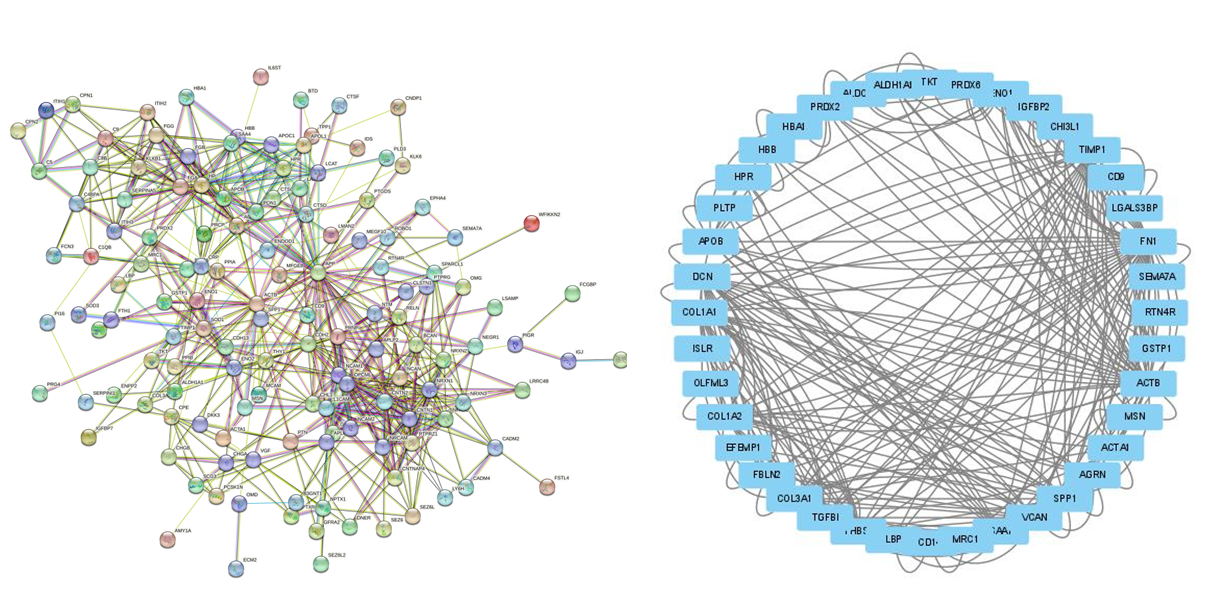
**

**Supplementary Figure 7:** Relations of survival and protein expression intensity.


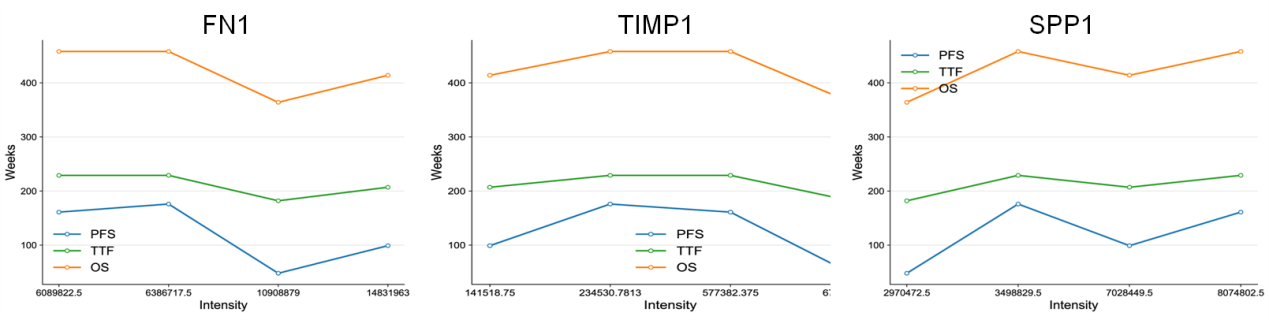


PFS, progress-free survival, the survival from NSCLC to LC; TTF, time to treatment failure, the survival from LC to deaths; OS, overall survival, the survival from NSCLC to deaths.
