# Supplementary material for: Cerebrospinal fluid exosomal protein alterations via proteomic analysis of NSCLC with leptomeningeal carcinomatosis
Source: J Neurooncol. 2023 Sep 1;164(2):367–76. doi: 10.1007/s11060-023-04428-x (PMC10522761; doi:10.1007/s11060-023-04428-x)
Supplement: Supplementary file 2 — Supplementary Material 2 [file 11060_2023_4428_MOESM2_ESM.docx]

**Supplementary Table 1** Trends and patterns of DEPs between LC vs. normal and LC vs. NSCLC

| Column | LC vs.NSCLC | LC vs. Normal | NSCLC vs. Normal | Gene name |
| --- | --- | --- | --- | --- |
| 1 | Up-regulation | Up-regulation | No difference | PIGR TKT MSN CD9 TIMP1 FCGBP MRC1 LBP ENO1 GSTP1 ACTA1 COL3A1 CTSC IGHG4 ACTB |
| 2 |  | Up-regulation | Down-regulation | QSOX1 |
| 3 | Down-regulation | Down-regulation | No difference | CHGA SPP1 |
| 4 |  | Down-regulation | Up-regulation | HBB PRDX2 HBA1 |
| 5 |  | Down-regulation | Down-regulation | NEGR1 RTN4R SEMA7A |
| 6 |  | Up-regulation | Up-regulation | ALDH1A1 LGLV10-54 HPR APOB |

Abbreviations: NSCLC, non-small cell lung cancer; LC, leptomeningeal carcinomatosis.

**Supplementary Table 2:** Target peptides for PRM analysis

| Master Protein Accessions | Gene Name | Annotated Sequence | Charge | m/z | RT [min] |
| --- | --- | --- | --- | --- | --- |
| P01033 | TIMP1 | GFQALGDAADIR | 2 | 617.315 | 33.937 |
| P01033 | TIMP1 | EPGLCTWQSLR | 2 | 673.830 | 36.410 |
| P01033 | TIMP1 | FVYTPAMESVCGYFHR | 3 | 655.299 | 44.373 |
| P01033 | TIMP1 | SEEFLIAGK | 2 | 497.266 | 29.094 |
| P01833 | PIGR | ILLNPQDK | 2 | 470.777 | 21.788 |
| P02751 | FN1 | STTPDITGYR | 2 | 555.775 | 24.461 |
| P02751 | FN1 | QYNVGPSVSK | 2 | 539.780 | 18.863 |
| P02751 | FN1 | EATIPGHLNSYTIK | 3 | 515.275 | 27.791 |
| P02751 | FN1 | EATIPGHLNSYTIK | 2 | 772.409 | 27.818 |
| P02751 | FN1 | YEVSVYALK | 2 | 536.290 | 33.059 |
| P06733 | ENO1 | IGAEVYHNLK | 3 | 381.877 | 20.123 |
| P06733 | ENO1 | VNQIGSVTESLQACK | 2 | 817.414 | 30.996 |
| P06733 | ENO1 | TIAPALVSK | 2 | 450.282 | 21.907 |
| P10451 | SPP1 | GDSVVYGLR | 2 | 483.256 | 25.979 |
| P10451 | SPP1 | YPDAVATWLNPDPSQK | 2 | 901.441 | 44.944 |
| P10451 | SPP1 | ISHELDSASSEVN | 2 | 694.320 | 20.930 |
| P22897 | MRC1 | SCVSLNPGK | 2 | 481.242 | 17.291 |
| P22897 | MRC1 | IYGTTDNLCSR | 2 | 650.304 | 23.287 |
| P22897 | MRC1 | FQWHEAETYCK | 3 | 500.219 | 26.096 |

**Supplementary Table 3:** Quantitative results of proteins after parallel reaction monitoring

| Gene Name | NSCLC/normal group | P | LC/normal group | P | LC/NSCLC | P |
| --- | --- | --- | --- | --- | --- | --- |
| TIMP1 | 3.141 | 0.071 | 1.345 | 0.514 | 0.428 | 0.134 |
| PIGR | 57.965 | 0.102 | 8.352 | 0.218 | 0.144 | 0.150 |
| FN1 | 0.600 | 0.268 | 1.911 | 0.113 | 3.185 | 0.029^*^ |
| ENO1 | 9.889 | 0.137 | 2.017 | 0.411 | 0.204 | 0.188 |
| MRC1 | 3.140 | 0.134 | 4.478 | 0.172 | 1.426 | 0.619 |
| SPP1 | 0.859 | 0.834 | 2.325 | 0.060 | 2.707 | 0.128 |

Abbreviations: NSCLC, non-small cell lung cancer; LC, leptomeningeal carcinomatosis; *, *P*＜0.05.

**Supplementary Table 4:** Concentration of exosomal protein in PRM

| Group | Normal group | | | | LC group | | | | NSCLC group | | | |
| --- | --- | --- | --- | --- | --- | --- | --- | --- | --- | --- | --- | --- |
| Patient number | 1 | 2 | 3 | 4 | 1 | 2 | 3 | 4 | 1 | 2 | 3 | 4 |
| Concentration of exosome protein (μg/μl) | 0.29 | 0.17 | 0.42 | 0.23 | 0.42 | 0.47 | 0.47 | 1.04 | 0.35 | 0.15 | 0.15 | 0.48 |
| Total volume (μl) | 550 | 500 | 250 | 250 | 280 | 310 | 260 | 200 | 460 | 500 | 300 | 100 |
| Total amount (μl) | 159.5 | 85.0 | 105.0 | 57.5 | 117.6 | 145.7 | 122.2 | 208.0 | 161.0 | 75.0 | 45.0 | 48.0 |
| Total CSF (ml) | 4.9 | 4.8 | 4.0 | 1.3 | 2.5 | 2.3 | 3.5 | 0.7 | 1.5 | 1.5 | 0.8 | 1.2 |

Abbreviations: NSCLC, non-small cell lung cancer; LC, leptomeningeal carcinomatosis; PRM, parallel reaction monitoring.
